# The environmental and social stress perception in autism spectrum disorder – focusing on stress coping mechanisms and gastrointestinal manifestations

**DOI:** 10.1192/j.eurpsy.2023.1943

**Published:** 2023-07-19

**Authors:** I. M. Balmus, M. A. Robea, R. Lefter, A. Ciobica, L. Gorgan, C. Stanciu, A. Trifan

**Affiliations:** 1 Department of Exact Sciences and Natural Sciences, Institute of Interdisciplinary Research; 2 Doctoral School of Biology, Faculty of Biology, Alexandru Ioan Cuza University of Iasi; 3 Center of Biomedical Research, Romanian Academy, Iasi Branch; 4 Department of Biology, Faculty of Biology, Alexandru Ioan Cuza University of Iasi; 5 Department of Gastroenterology, Faculty of Medicine, Gr. T. Popa University of Medicine and Pharmacy, Iasi, Romania

## Abstract

**Introduction:**

Autism spectrum disorder (ASD) is currently defined as persistent deficits in social communication and social interaction additional to repetitive behavioural patterns, and restrictive interests or activities. Several studies suggested that ASD is accompanied by defective perception and altered response to the environmental factors, including environmental and social stress. In this context, frequent reports addressed the manifestation of different stress-related behavioural and physiological patterns, such as restless leg syndrome, migraine, and functional gastrointestinal disorders (FGIDs). Thus, a major problem in ASD patients’ management could be related to the exacerbated effects of stress.

**Objectives:**

In this study, we aimed to find a correlation between the stress perception and response and the manifestation of some neurological and FGIDs in ASD.

**Methods:**

The main scientific databases were screened for studies describing the effects of stress in autism patients and animal models. Exclusion criteria: (1) studies not written in English language; (2) not available as full text; (3) not describing stress response; and/or (2) functional gastrointestinal manifestations in autism.

**Results:**

The repetitive behaviours have a heterogenous pattern in both severity and manifestations, varying from repetitive motor movements and inflexible adherence to routines to hypo- or hyper-reactivity to exterior stimuli. Moreover, some studies describe repetitive behaviours as altered stress coping mechanisms meant to relieve anxious states – one of the main stress axis activation effects. We recently described some significantly reported FGID-like manifestations in ASD that could be the result of various abnormalities in the brain – gut interaction, such as impaired parasympathetic activity and increased endocrine stress response. In this context, there could be a correlation between the altered perception and response to stress and the FGID-like manifestations, as we previously described the stress axis implication in one of the most common FGID, irritable bowel syndrome – which is also frequently reported in ASD cases.

**Conclusions:**

In ASD, the perception and response to environmental and social stress could be impaired. Thus, impaired stress coping mechanisms, defective stress axis, and altered behavior could lead to stress-specific manifestations, such as restless leg syndrome, migraine, and irritable bowel syndrome.

Funding: *B. I.-M. and *R. M.-A. are supported by the Project POCU/993/6/13/153322 ”Suport educațional și formativ pentru doctoranzi și tineri cercetători în pregătirea inserției în piața muncii” of the European Social Fund through the Human Capital Operational Program

**Disclosure of Interest:**

I. M. Balmus Grant / Research support from: B. I.-M. is supported by the Project POCU/993/6/13/153322 ”Suport educațional și formativ pentru doctoranzi și tineri cercetători în pregătirea inserției în piața muncii” of the European Social Fund through the Human Capital Operational Program., M. A. Robea Grant / Research support from: R. M.- A. is supported by the Project POCU/993/6/13/153322 ”Suport educațional și formativ pentru doctoranzi și tineri cercetători în pregătirea inserției în piața muncii” of the European Social Fund through the Human Capital Operational Program., R. Lefter: None Declared, A. Ciobica: None Declared, L. Gorgan: None Declared, C. Stanciu: None Declared, A. Trifan: None Declared

